# Development of a phantom for assessing the precision of setup in skin mark‐less surface‐guided radiotherapy

**DOI:** 10.1002/acm2.14381

**Published:** 2024-05-02

**Authors:** Masahide Saito, Koji Ueda, Hikaru Nemoto, Yoshiko Onishi, Hidekazu Suzuki, Toshihiro Suzuki, Naoki Sano, Takafumi Komiyama, Kan Marino, Hiroshi Onishi

**Affiliations:** ^1^ Department of Radiology University of Yamanashi Yamanashi Japan; ^2^ Kasugai General Rehabilitation Hospital Yamanashi Japan

**Keywords:** breast, face, mark‐less, phantom, SGRT

## Abstract

**Background:**

Surface‐guided radiotherapy (SGRT) is adopted by several institutions; however, reports on the phantoms used to assess the precision of the SGRT setup are limited.

**Purpose:**

The purpose of this study was to develop a phantom to verify the accuracy of the irradiation position during skin mark‐less SGRT.

**Methods:**

An acrylonitrile butadiene styrene (ABS) plastic cube phantom with a diameter of 150 mm on each side containing a dummy target of 15 mm and two types of body surface‐shaped phantoms (breast/face shape) that could be attached to the cube phantom were fabricated. Films can be inserted on four sides of the cubic phantom (left, right, anterior and posterior), and the center of radiation can be calculated by irradiating the dummy target with orthogonal MV beams. Three types of SGRT using a VOXELAN‐HEV600M (Electronics Research&Development Corporation, Okayama, Japan) were evaluated using this phantom: (i) SGRT_CT_—a SGRT set‐up based solely on a computed tomography (CT)‐reference image. (ii) SGRT_CT_ + CBCT—a method where cone beam computed tomography (CBCT) matching was performed after SGRT_CT_. (iii) SGRT_Scan_—a resetup technique using a scan reference image obtained after completing the (ii) step.

**Results:**

Both the breast and face phantoms were recognized in the SGRT system without problems. SGRT_Scan_ ensure precision within 1 mm/1° for breast and face verification, respectively. All SGRT methods showed comparable rotational accuracies with no significant disparities.

**Conclusions:**

The developed phantom was useful for verifying the accuracy of skin mark‐less SGRT position matching. The SGRT_Scan_ demonstrated the feasibility of achieving skin‐mark less SGRT with high accuracy, with deviations of less than 1 mm. Additional research is necessary to evaluate the suitability of the developed phantoms for use in various facilities and systems. This phantom could be used for postal surveys in the future.

## 1 INTRODUCTION

Image‐guided radiotherapy (IGRT) has become an indispensable technology for high‐precision radiotherapy, and recent surveys have shown that several institutions are employing this technology.^1^, ^2^, ^3^ Although conventional IGRT methods are generally performed using X‐rays, the effective management of radiation doses at the time of imaging is a problematic issue. Therefore, the American Association of Physicists in Medicine (AAPM) task group 75 report emphasizes the need to consider the specific radiation dosage received by each patient during IGRT.^4^ In recent years, surface‐guided radiotherapy (SGRT) has been introduced in some institutions, which is a positioning technique for radiotherapy using body surface images acquired with optical devices. In the current situation, the AAPM task group report 302 is published as a guideline for SGRT, and it mentions guidelines regarding positional accuracy and provides a set of recommendations.^5^

SGRT can be used for patient positioning without radiation exposure as well as for respiratory motion management, such as the deep inspiration breath‐hold (DIBH) method. The use of SGRT has been documented as a means to assist with patient positioning without the need to use skin marks (tattoos). It has been reported that more than half of patients show an uncomfortable with skin marks during radiotherapy.^6^ Particularly for the treatment of breast cancer, it has been observed that many patients are averse to conventional skin marks and tattoos.^7^ Consequently, several studies on the use of skin mark‐less treatment using SGRT have been conducted,^8^, ^9^, ^10^ taking into account the emotional discomfort experienced by the patients. However, there are concerns about the accuracy of the method in terms of patient positioning when compared with conventional methods using skin marks, tattoos, or X‐ray IGRT (such as CBCT).

Few phantoms have been reported for assessing the precision of SGRT irradiation. Bry et al. developed a comprehensive phantom that incorporates a gel dosimeter into the head region.^11^ Kadman et al. designed a deformable phantom specifically for the verification of SGRT accuracy.^12^ Covington et al. introduced an economical method for assessing the SGRT accuracy using an EPID.^13^ Numerous evaluations employing whole‐body phantoms have also been carried.^14^ It is likely that both a head and shoulder phantom (model 136500, CIRS, Norfolk, VA) and a thorax phantom (model 036S, CIRS, Norfolk, VA, USA) were utilized for comprehensive end‐to‐end testing.

However, these phantoms are often facility‐specific measurement systems and are not suitable for assessing the setup accuracy of multicenter SGRT systems. For example, the IROC head and neck phantom not only features a complex facial geometry but also incorporates a glass dosimeter or film encased in a precise location.^15^ However, its primary function is to evaluate dose distribution near the target area, and its inability to assess alignment accuracy across six axes makes it unsuitable for applications that prioritize setup precision. Although several phantoms for postal audit tests in conventional IGRT using X‐rays have been developed and applied in clinical trials that enable irradiation position accuracy assessment to be performed,^16^, ^17^, ^18^ no such phantoms have been developed for SGRT. The postal audit function may be an important factor in equalizing the quality of treatment for SGRT. Therefore, this study aimed to develop a phantom for verifying the accuracy of irradiation positions in skin mark‐less SGRT, which can be utilized in future postal surveys.

## 2 METHODS

### 2.1 Phantom characteristics

**FIGURE 1:**
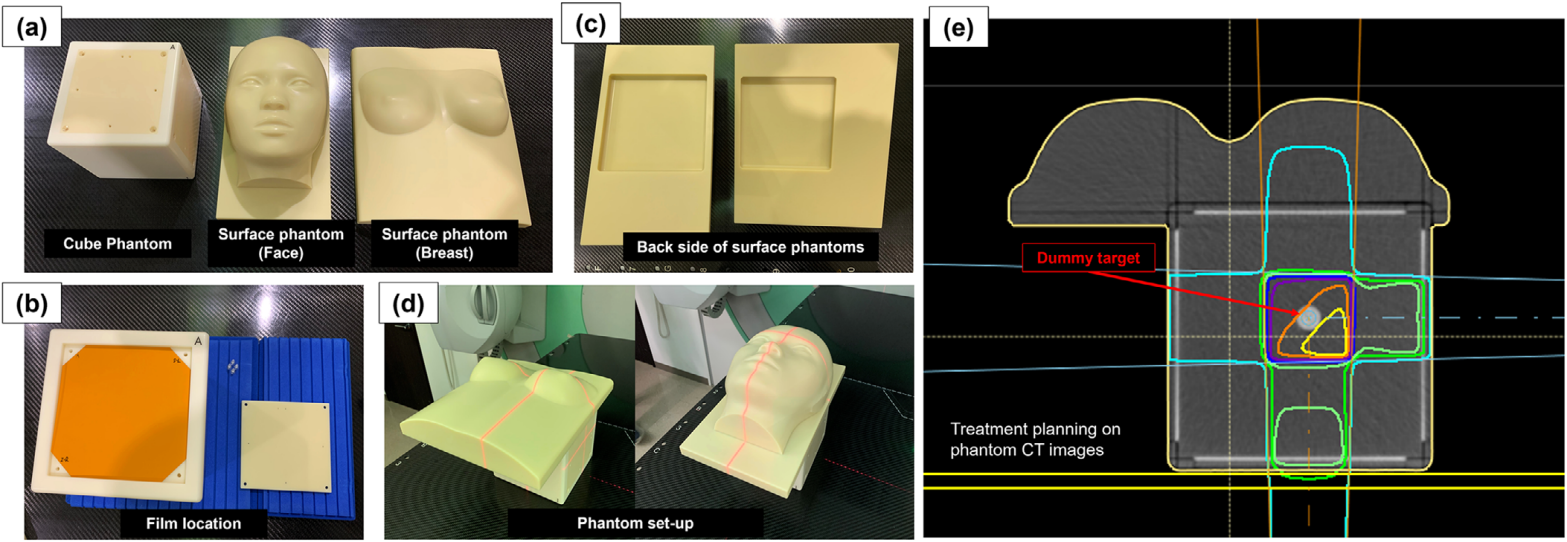
Visual representation of body surface phantoms (a) Left: cube phantom, center: face surface phantom, right: breast surface phantom; (b) Film location inside the cube phantom; (c) Back side of both surface phantoms (left: face phantom, right: breast phantom). The depth of the groove is 10 mm; (d) Overview of phantom set‐up (left: face, right: breast); (e) Sample of treatment planning based on phantom CT images. The isocenter was positioned at the center of gravity of the dummy target within the cube phantom. Irradiation was executed from gantry angles of 180° and 90° with 5 × 5 cm fields.

Figure 1a illustrates the visual representation of the SGRT phantom. The phantom consists of two components. First, a cubic phantom was made of Acrylonitrile Butadiene Styrene (ABS) plastic with dimensions of 150 × 150 × 150 mm. A dummy target with a diameter of 15 mm was placed inside the phantom (Figure 1e). Furthermore, the phantom was equipped with four gold fiducial markers, each 1.5 mm in diameter with a physical density of 19.3 g/cm^3^ (disposable gold marker, Olympus Corporation, Tokyo, Japan), facilitating the implementation of various X‐ray IGRT techniques. Four 140 mm × 140 mm planes on the left, right, anterior, and posterior sides are fixed with screws at the four corners. These screws can be removed to allow insertion of a processed Gafchromic™ RTQA2 film (Figure 1b). In addition, the plates had four small holes through which the reference points could be plotted after the film was inserted.

The center and right of Figure 1a show the surface‐shaped phantoms for the face and breast, respectively. Figure 1c shows the back sides of these phantoms, where a groove measuring 150 × 150 × 10 mm was machined, allowing these phantoms to fit precisely into the cubic phantom. Figure 1d shows the setup of each phantom in its assembled state.

### 2.2 SGRT system

In this study, a VOXELAN‐HEV600M (Electronics Research&Development Corporation, Okayama, Japan) installed in the same room as the Elekta Synergy System was used as the SGRT system. The VOXELAN system hung from the ceiling. Laser scans from the projection windows on either side of the device were recorded using a CCD camera at the center.

The distance from the VOXELAN to the radiation isocenter was 1700 mm, and the field of view (FOV) was 600 × 450 × 600 mm. Two methods for generating a reference surface image exist: one involves the use of CT (CT reference) while other involves employing a scanned image (scan reference) as the reference image. The resolution of the output reference image from the software was 0.75 mm for both the CT reference and the scan reference. Further details on the VOXELAN are provided in a previous report.^14^

### 2.3 Evaluation workflow

**FIGURE 2:**
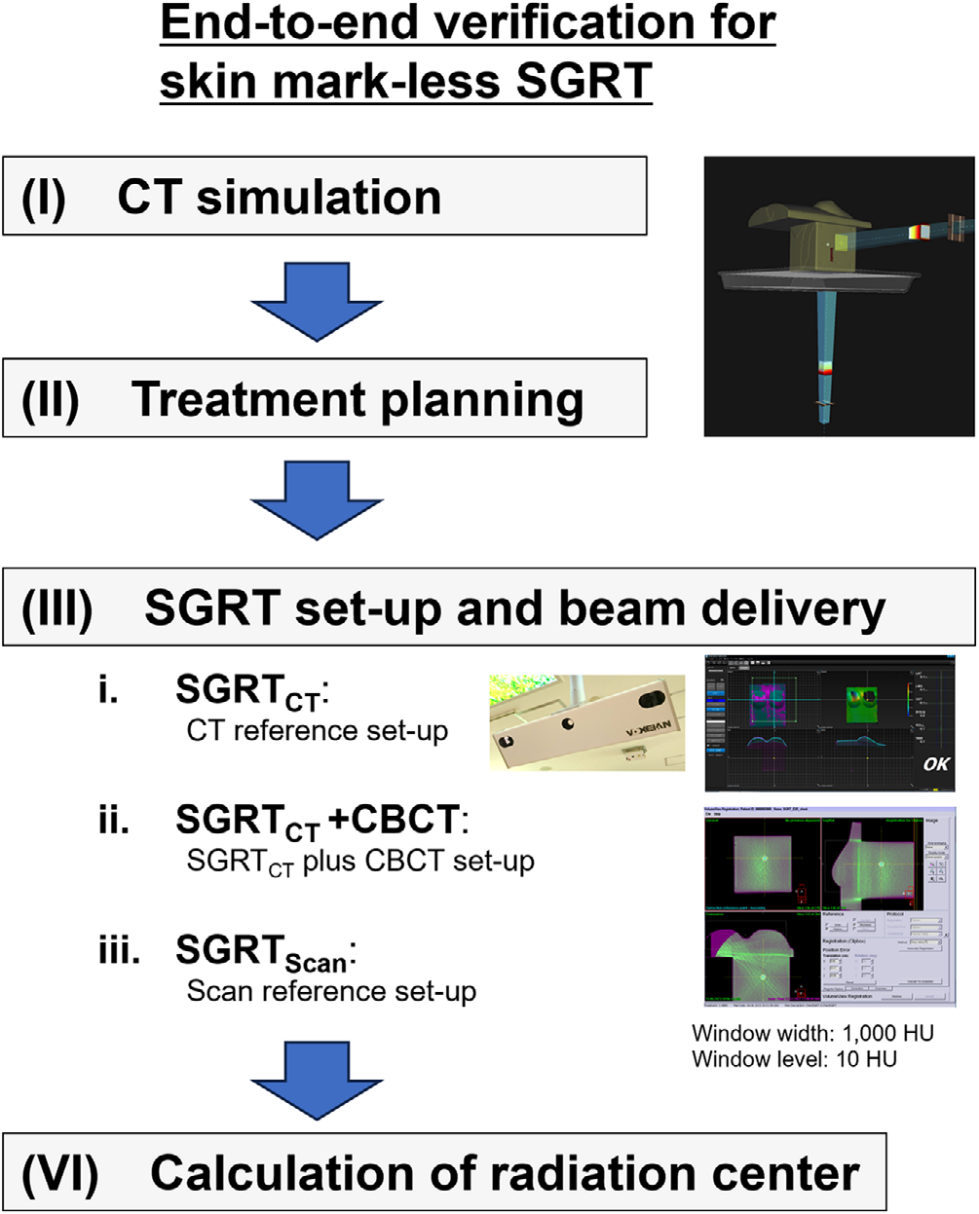
Flowchart of the end‐to‐end test (I) CT simulation was conducted for each phantom. (II) Treatment planning entailed perpendicular port irradiation to the dummy target inside the cube phantom. (III) Three SGRT set‐up patterns were executed: (i) SGRT_CT_—a set‐up based solely on CT‐reference. (ii) SGRT_CT_ + CBCT—a method where CBCT matching was performed after SGRT_CT_. (iii) SGRT_Scan_—a re‐set‐up technique using a scan reference obtained after completing step (ii). (IV) The radiation center was determined using films taken during irradiation.

The workflow of this study is shown in Figure 2. This workflow was implemented for both breast‐ and face‐shaped surface phantoms. Experienced radiological technologists and medical physicists conducted the phantom setup and image matching three times for each SGRT method, after which the mean values and variations were assessed. Statistical analyses were performed using the ANOVA for three groups and t‐test for two groups (JMP Pro ver.17, SAS Institute, Cary, NC). The specifics of the workflow are as follows:

- CT simulation

After the RTQA2 films were inserted on all four sides of the cubic phantom, a surface‐shaped phantom was affixed to it, and a CT scan was conducted for treatment planning. Treatment‐planning CT images were acquired using an Aquilion LB (Canon Medical Systems, Otawara, Japan). The imaging was set to a 55‐cm FOV, and the device was operated at a tube voltage of 120 kVp and a current of 250 mA. The voxel dimensions were approximately 1 × 1 × 2.0 mm.

(ii) Treatment planning

The process of 3D treatment planning was conducted based on the CT images. The beam isocenter was positioned at the center of gravity of the dummy target. Perpendicular port irradiation was then performed. The gantry angles were set to 180° and 90°. The monitor units were set to 600 for each beam. The treatment planning system used in this study was the RayStation ver10.A (RaySearch Lab, Sweden). The dose calculation algorithm employed in this study was collapsed cone convolution with a calculation grid size of 2 mm.

(iii) SGRT set‐up and beam delivery

Three different SGRT setups ((i) SGRT_CT_, (ii) SGRT_CT_ +CBCT, and (iii) SGRT_Scan_) were implemented in this study. In our institution, we typically use SGRT_CT_. If changes in body shape make SGRT_CT_ unsuitable, we then consider using SGRT_Scan_ as needed. (i) SGRT_CT_ employs only SGRT with CT as a reference for setup and irradiation. Treatment‐planning CT images were imported into the VOXELAN software to generate a reference body surface image. Using this reference, the phantom setup was adjusted until the SGRT device displayed values within the range of 1 mm/1°. Perpendicular port irradiation was then performed using this setup. (ii) SGRT + CBCT is a technique that involves performing a setup using the CT reference, then acquiring a CBCT image and making a correction in that state. Based on the acquired CBCT images, position matching was performed, and the setup was operated until the indicated value of the IGRT device was within 1 mm/1°. X‐ray volume imaging (XVI®) system ver. 5.0 (Elekta AB, Crawley, UK) attached to the Elekta Synergy was used for the CBCT‐based IGRT. Following this setup, perpendicular port irradiation was conducted, and a scan reference was acquired using the SGRT device immediately before irradiation. (iii) SGRT_Scan_ employs only the SGRT with the scan reference obtained after CBCT matching (i.e., after the above procedure) for the setup and irradiation. The scan reference was read by the SGRT system and the phantom setup was based on the scan reference, which was adjusted until the SGRT device displayed values within a 1 mm/1° range. Perpendicular port irradiation was then performed using this setup.

(iv) Calculation of radiation center

**FIGURE 3:**
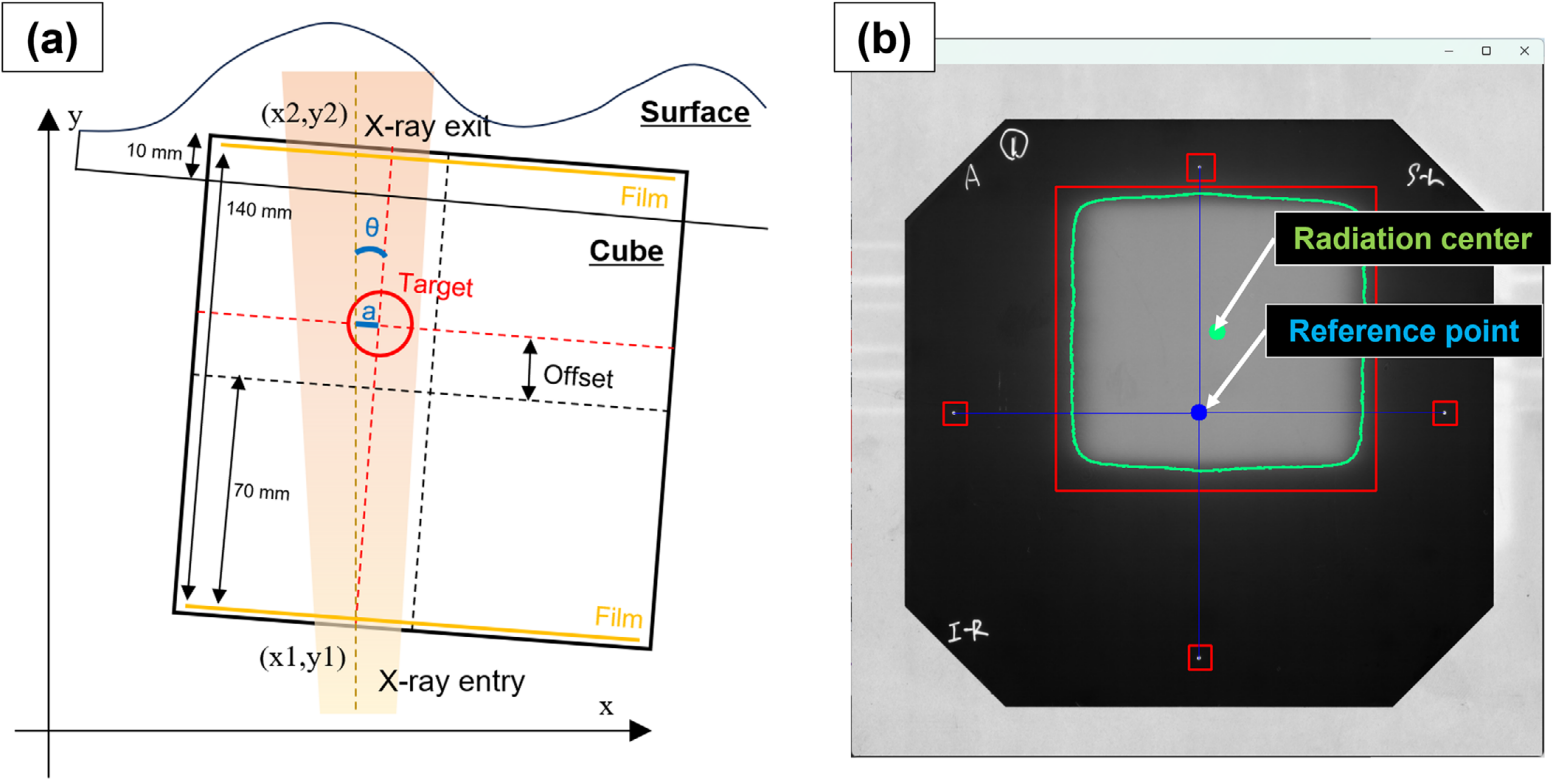
Schematic diagram of film analysis (a) Illustration detailing the calculation of the translation error a and rotation error θ. Coordinates $x_{1}$, $y_{1}$ represent the film's position on the X‐ray entry side, while $x_{2}$, $y_{2}$ denote the film's position on the X‐ray exit side. The distance to the contralateral film was 140 mm. The offset refers to the positional shift of the spherical target from the phantom center along other axes. (b) Analysis window displaying the post‐irradiation film. Markings on all four sides are automatically recognized, and the reference point was established. The radiation center was determined through edge detection in binary image processing.

The RTQA2 films were loaded using an EPSON ES‐GS11000 instrument at a resolution of 300 dpi. The film was placed in the center of the scanner and scans were carried out three times. Then, the data was saved in JPEG format. The accuracy of the irradiation position along the three axes of translation and rotation was calculated using almost the same method as described in a previous study.^16^ One aspect that sets this method apart from previously reported methods is the change in the gantry angle to 180° to avoid incidence on the body surface. Before the experiments, the accuracy of the entire system was confirmed by subjecting it to error irradiation with a 3 mm translation error and a 1 degree rotation error. Figure 3a shows a schematic of the indices used for the calculations. The translational axis misalignment, denoted as “a,” is defined as follows:

$$\begin{array}{ccc} & & a\left[mm\right]=\left[x_{1}+\left(\frac{\left(x_{2}-x_{1}\right)}{140}\right)\cdot \left(Offset+70\right)\right]\;-x_{dcm}\\ & & -x_{shift} \end{array}$$

where $x_{1}$ represents the positional coordinates of the X‐ray entry side and $x_{2}$ represents the positional coordinates of the X‐ray exit side. The offset refers to the positional shift of the spherical target from the phantom center along the other axes. $x_{dcm}$ is the correction value calculated from DICOM‐RT, and $x_{shift}$ is the known shift from the phantom center.

The rotation axis misalignment, denoted as “θ,” is defined as follows:

$$\theta \left[deg.\right]\;=\tan^{-1}\left(\frac{x_{2}-x_{1}}{140}\right)\cdot \frac{180}{\pi }\;-\theta_{dcm}$$

where $x_{1}$ represents the positional coordinates of the X‐ray entry side and $x_{2}$ represents the positional coordinates of the X‐ray exit side. $\theta_{dcm}$ is the correction value calculated from the CT images. These analyses were performed using an in‐house Python program (Figure 3b).

## 3 RESULTS

**FIGURE 4:**
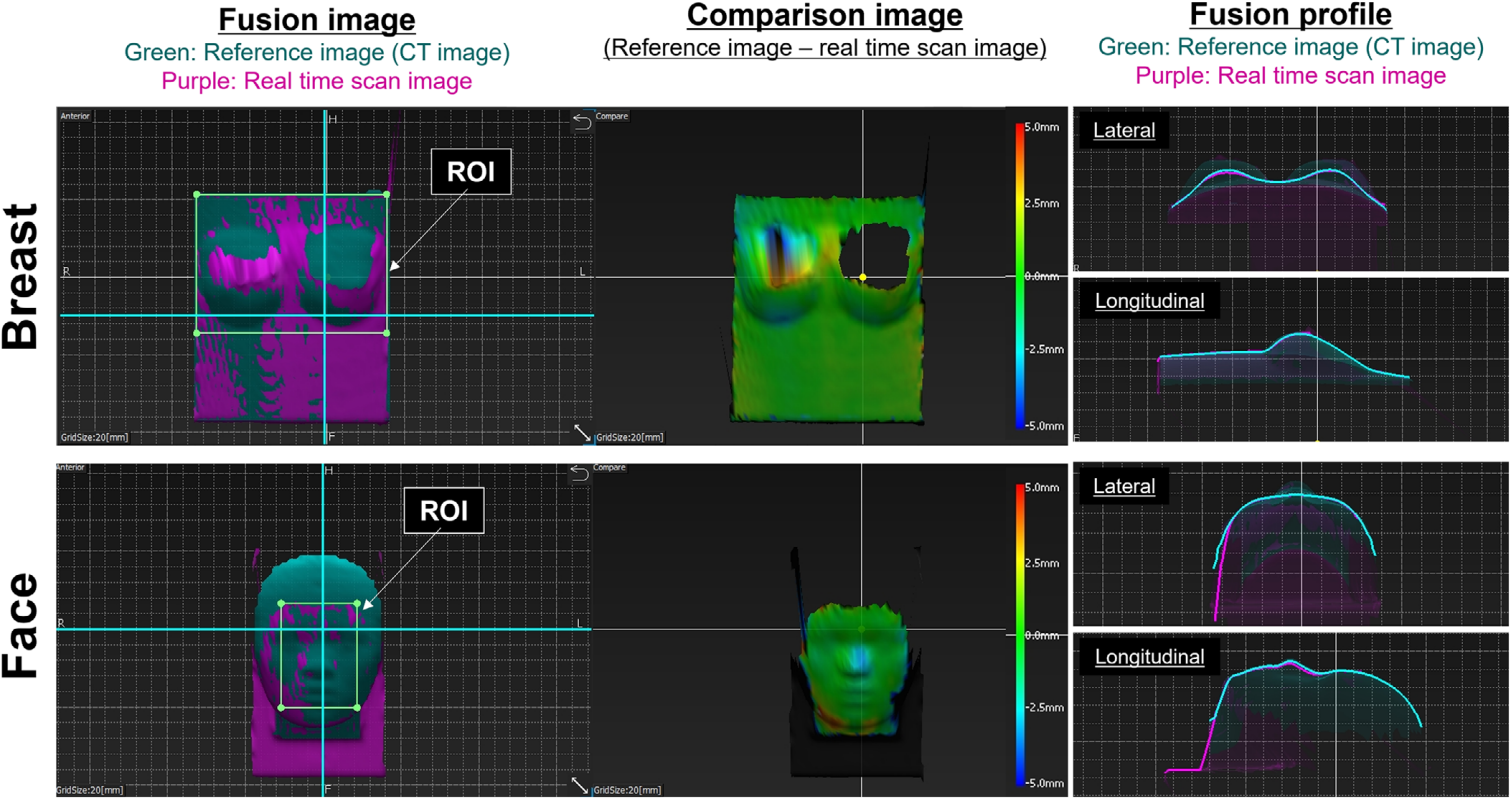
Examples of SGRT images featuring two surface phantoms are presented. The upper rows display results for the breast surface, while the lower rows focus on the face surface. The fusion images are depicted on the left. The middle section presents comparison images, with color bars indicating the positional error in relation to the reference image. The fusion profiles are presented on the right.

Typical surface images acquired using each SGRT technique are shown in Figure 4. Both the breast and face phantoms can be recognized in the SGRT system without problems. However, image loss occurred in areas where the camera had a blind spot.

The results of the film analysis are presented in Table 1. Regarding the translational axis for breast shape verification, the mean values (lateral (LR)/anterior‐posterior (AP)/superior‐inferior (SI), mm) for SGRT_CT_, SGRT_CT_+CBCT, and SGRT_Scan_ were −1.42/1.74/0.00, 0.01/0.39/−0.54, and −0.25/0.23/−0.26, respectively. Similarly, for another aspect of face shape verification, the values were −0.03/3.11/2.25, −0.12/0.35/−0.02, and −0.53/0.12/−0.39, respectively. For the breast phantom, when comparing SGRT_CT_ with SGRT_CT_+CBCT, SGRT_CT_ + CBCT demonstrated significantly better accuracy in the LR and AP directions. No significant differences were observed between the SGRT_CT_+CBCT and SGRT_Scan_. However, when comparing SGRT_CT_ and SGRT_Scan_, SGRT_Scan_ was found to be notably more precise in the AP direction. For the face phantom, when comparing SGRT_CT_ with SGRT_CT_+CBCT, SGRT_CT_ + CBCT demonstrated significantly better accuracy in the AP and SI directions. No significant differences were observed between the SGRT_CT_+CBCT and SGRT_Scan_. However, comparing SGRT_CT_ and SGRT_Scan_ revealed that SGRT_Scan_ was notably more precise in all translational directions.

**TABLE 1:** Accuracy of radiation center position for each SGRT method (mean ± standard deviation).

			Accuracy of radiation center position (mm or deg., *n* = 3)			*p*‐value			
							*t*‐test		
			SGRT_CT_	SGRT_CT_+CBCT	SGRT_Scan_	ANOVA (3‐groups)	SGRT_CT_ vs. SGRT_CT_+CBCT	SGRT_CT_+CBCT vs. SGRT_Scan_	SGRT_CT_ vs. SGRT_Scan_
Breast	Trans (mm)	LR	−1.42 ± 0.19	0.01 ± 0.21	−0.25 ± 0.33	<0.01*	0.04*	0.47	0.05
		AP	1.74 ± 0.31	0.39 ± 0.37	0.23 ± 0.10	<0.01*	0.03*	0.52	0.01*
		SI	0.00 ± 0.25	−0.54 ± 0.19	−0.26 ± 0.66	0.49	0.11	0.50	0.64
	Roll (deg.)	Pitch	0.55 ± 0.03	0.98 ± 0.26	0.85 ± 0.13	0.10	0.12	0.56	0.09
		Yaw	0.08 ± 0.29	−0.23 ± 0.07	−0.14 ± 0.50	0.66	0.33	0.82	0.40
		Roll	−0.02 ± 0.06	0.04 ± 0.04	−0.08 ± 0.07	0.19	0.52	0.20	0.37
Face	Trans (mm)	LR	−0.03 ± 0.35	−0.12 ± 0.01	−0.53 ± 0.27	0.20	0.74	0.16	0.04*
		AP	3.11 ± 0.14	0.35 ± 0.20	0.12 ± 0.19	<0.01*	<0.01*	0.06	<0.01*
		SI	2.25 ± 0.18	−0.02 ± 0.30	−0.39 ± 0.56	<0.01*	0.02*	0.38	0.03*
	Roll (deg.)	Pitch	0.11 ± 0.02	0.45 ± 0.41	0.19 ± 0.06	0.38	0.34	0.50	0.25
		Yaw	−0.71 ± 0.02	−0.30 ± 0.20	−0.13 ± 0.30	0.08	0.12	0.43	0.12
		Roll	−0.19 ± 0.07	−0.17 ± 0.11	−0.24 ± 0.02	0.68	0.55	0.47	0.51

*p‐value < 0.05.

For the rotational axis for breast shape verification, the mean values (pitch/roll/yaw, deg.) for SGRT_CT_, SGRT_CT_+CBCT, and SGRT_Scan_ were 0.55/0.08/−0.02, 0.98/−0.23/0.04, and 0.85/−0.14/−0.08, respectively. Similarly, for another aspect of face shape verification, the values were 0.11/−0.71/−0.19, 0.45/−0.30/−0.17, and 0.19/−0.13/−0.24. In comparison, the breast and face phantoms exhibited no significant differences in rotational accuracy among the three SGRT methods.

## 4 DISCUSSION

SGRT is a pivotal technology in the field of minimally invasive radiotherapy and has recently garnered significant attention for its applications in DIBH and skin mark‐less radiotherapy. However, to the best of our knowledge, no study has reported the development of a postal audit phantom for SGRT. This study was the first phantom development and evaluation. The development of such a phantom is crucial for ensuring consistent matching accuracy using SGRT across various institutes.

The primary measurement uncertainties of this method were discussed in a previous paper, which included translation (APLR: 0.50 mm, SI: 0.75 mm) and rotation (0.33°) uncertainties.^16^ These uncertainties arise from manufacturing errors, discrepancies in setting the reference mark position, determination of the radiation field center, and DICOM analysis. However, in this study, in addition to the previously mentioned uncertainties, there is a potential for errors arising from mistakes in the assembly of the surface‐shaped phantom. While quantitatively assessing this factor is challenging, CT images show minimal gaps between the surface and cube phantoms, suggesting minimal displacement when the phantom is assembled correctly. Thus, it is believed that the phantom developed in this study can be utilized for postal surveys, offering accuracy in line with prior literature.

In the present study, the results obtained using the SGRT_CT_ tended to be worse than the results obtained using the SGRT_CT_+CBCT, and SGRT_Scan_. These errors may have originated from the resolution of the treatment planning CT, as well as from inaccuracies introduced by the reconstruction method utilized by the VOXELAN software for body surface creation. In this study, a slice thickness of 2 mm was employed. This was comparable to previous reports^14^ and was attributed to the reliance of final matching accuracy on the resolution of the original CT images and the image processing performed inside the program when generating reference pictures from CT images. Furthermore, VOXELAN features its proprietary body surface reconstruction software, which may have also impacted the accuracy. Since it does not align with the AAPM TG302 criteria within the current experimental setup, we believe it cannot be directly utilized as is. Previous reports have shown that the positional error can be effectively reduced to less than 3 mm without X‐ray‐based IGRT.^19^ However, to minimize latent errors as much as possible in real‐world clinical practice, it is preferable to perform SGRT using SGRT_Scan_ in conjunction with CBCT, as suggested by the results of this study. In addition, according to the AAPM TG302 report, the static localization accuracy and isocenter coincidence with other imaging modalities must be controlled to <2 mm (<1 mm for SRS/SBRT) as the quality assurance for SGRT.^5^ Therefore, this study was conducted appropriately to comply with these requirements.

One limitation of this study was the single‐center evaluation of the measurements. Especially, careful feedback should be obtained from multi‐facilities on the assembly of the phantom. Another limitation concerns the detection precision of the SGRT device, which might be compromised due to the tumor's deeper anatomical location of this phantom relative to the clinical case. Therefore, it remains to be determined whether this phantom can be employed across multiple facilities and systems.

## 5 CONCLUSION

In this study, we verified the accuracy of skin mark‐less SGRT position matching using a newly developed phantom for both breast and face shapes. For SGRT_Scan_, skin mark‐less irradiation was achieved with a high accuracy, with deviations of less than 1 mm. However, potential matching errors were highlighted for SGRT_CT_. This phantom could be used for postal surveys in the future. Further studies are required to assess the applicability of the developed phantoms across multiple facilities and systems.

## AUTHOR CONTRIBUTIONS

*Study conception and design*: Masahide Saito. *Acquisition of data*: Masahide Saito, Koji Ueda, Hikaru Nemoto, Yoshiko Onishi, Hidekazu Suzuki, Toshihiro Suzuki, and Naoki Sano. *Analysis and interpretation of data*: Masahide Saito. *Drafting of manuscript*: Masahide Saito, and H.O. *Critical revision*: Masahide Saito, Takafumi Komiyama, Kan Marino, and Hiroshi Onishi.

## CONFLICT OF INTEREST STATEMENT

This study was supported by joint research with APEX Medical Inc. and Hamano Engineering.

## Data Availability

The data that support the findings of this study are available from the corresponding author upon reasonable request.
